# Spontaneous perception of numerosity in humans

**DOI:** 10.1038/ncomms12536

**Published:** 2016-08-24

**Authors:** Guido Marco Cicchini, Giovanni Anobile, David C. Burr

**Affiliations:** 1 Institute of Neuroscience, National Research Council, 56124 Pisa, Italy; 2 Department of Neuroscience, Psychology, Pharmacology and Child Health, University of Florence, 50121 Florence, Italy; 3 Department of Developmental Neuroscience, Stella Maris Scientific Institute, 56018 Pisa, Italy; 4 School of Psychology, University of Western Australia, 6009 WA Perth, Australia

## Abstract

Humans, including infants, and many other species have a capacity for rapid, nonverbal estimation of numerosity. However, the mechanisms for number perception are still not clear; some maintain that the system calculates numerosity via density estimates—similar to those involved in texture—while others maintain that more direct, dedicated mechanisms are involved. Here we show that provided that items are not packed too densely, human subjects are far more sensitive to numerosity than to either density or area. In a two-dimensional space spanning density, area and numerosity, subjects spontaneously react with far greater sensitivity to changes in numerosity, than either area or density. Even in tasks where they were explicitly instructed to make density or area judgments, they responded spontaneously to number. We conclude, that humans extract number information, directly and spontaneously, via dedicated mechanisms.

## Abstract (web summary)

Humans as well as many other species have the ability to perceive the number of items, numerosity, but the mechanism by which this is achieved is unclear. Here the authors provide strong evidence in support of a spontaneous perception of numerosity without reliance on density and area estimation.

## Graphical abstract

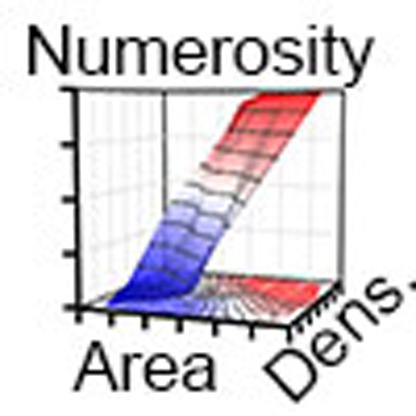

Perceiving the number of objects is a fundamental survival skill for many animal species^1^,^2^. Humans are capable of estimating numerosity very early during development, probably even at birth^3^. Precision for numerosity improves steadily with age up to about 30 years^4^ and, importantly, predicts mathematical proficiency^5^,^6^,^7^. But how humans and animals estimate numerosity remains an open question. Both animal^8^,^9^,^10^,^11^ and human^12^,^13^ research points to dedicated neural structures for numerosity perception, but it remains an open question as to whether observers estimate numerosity *per se*, or rather infer it from other visual quantities that covary with numerosity, such as density. In part the debate has revolved around the whether numerosity estimation operates over unsegmented visual textures^14^,^15^,^16^ or whether the scenes need first to be segmented into objects^17^,^18^,^19^,^20^,^21^. There has been a good deal of evidence for both sides of the debate^15^,^16^,^22^,^23^,^24^,^25^,^26^,^27^,^28^,^29^,^30^,^31^, with no definitive resolution.

We have recently suggested that both number and texture-like mechanisms may operate, depending on the density of the displays^32^. At low to moderate densities, where the items can be segregated, there is clear evidence for numerosity mechanisms; at higher densities, where the objects become crowded, texture-like mechanisms may be at work. The evidence for the different mechanisms comes largely from different psychophysical laws operating at different densities, Weber's law at the moderate densities where the objects can be segregated, and a square root law at higher densities^22^. There is also a clear dependence of eccentricity, implicating crowding-like mechanisms^33^.

But the basic question remains whether numerosity is sensed directly, via dedicated mechanism, or derived indirectly from density and area, as has been suggested^16^,^25^,^27^,^31^. To this end we created a two-dimensional (2D) space representing density, area and numerosity, and examined which dimensions human observers were most sensitive. The results show that at moderate densities, observers were far more sensitive to numerosity than to either density or area. Only at very high-dot densities did observers tend to use density and area information directly.

## Results

**Figure 1:**
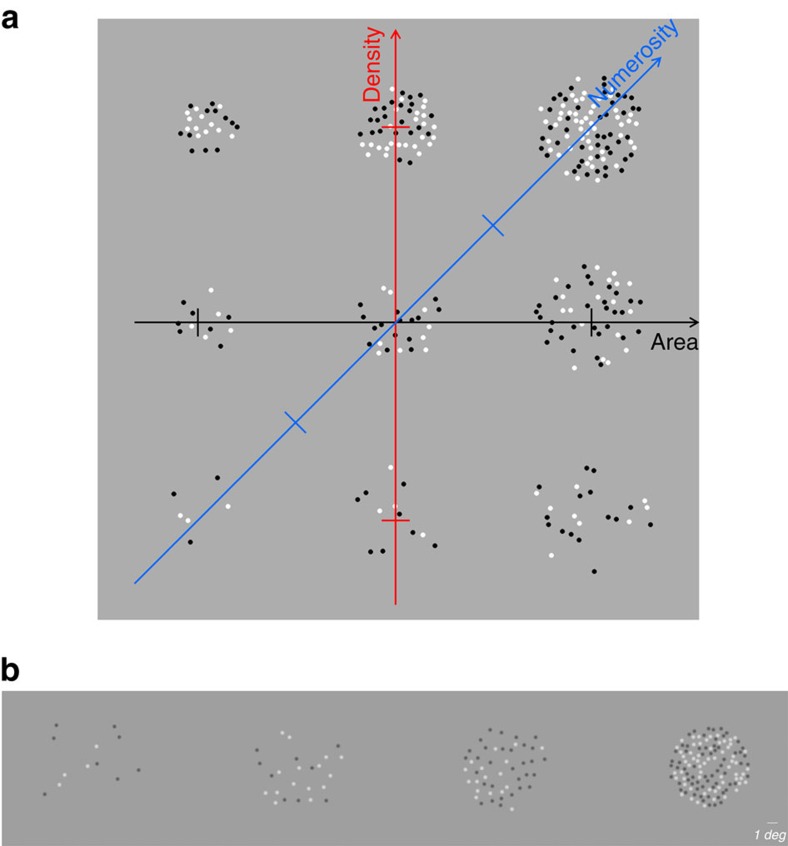
Area–density stimulus space. (**a**) Schematic illustration of the 2D space describing the stimuli used in this study. The origin is the ‘standard' stimulus for a specific condition, always of radius 3.6 degrees (area 40 sq degrees), with 12, 24, 32, 48, 64 or 128 dots (density ranged from 0.3 to 3.2 dots per deg^2^). The abscissa plots relative stimulus area, and the ordinate relative density. The positive diagonal represents relative number. Lines orthogonal to this diagonal have constant number. All axes are logarithmic: each tick shows an octave (base-two logarithm) interval, a doubling or halving of that quantity. Note this is a schematic illustration. (**b**) Example of the actual stimuli for numerosities 12, 24, 48 and 128.

The general procedure was to measure discrimination thresholds for stimuli that varied over two dimensions, area and density. Figure 1 shows an example of the stimulus space, on logarithmic axes, with the origin depicting the standard area and density of a particular condition, and the ticks on the axes showing octave changes (doubling or halving of that dimension). As numerosity is the product of area and density, it can be depicted in this logarithmic space as the +45° diagonal. We then measure discrimination thresholds in all directions within the space to determine the direction of maximum sensitivity, and relate that to the directions of area, density and number. The logic is similar to that of measuring Macadam ellipses in color-space^34^. Indeed the thresholds of experiment 1 are well described by ellipses, with short radii corresponding to the most sensitive direction in this space.

### Discrimination boundaries in the area–density space

**Figure 2:**
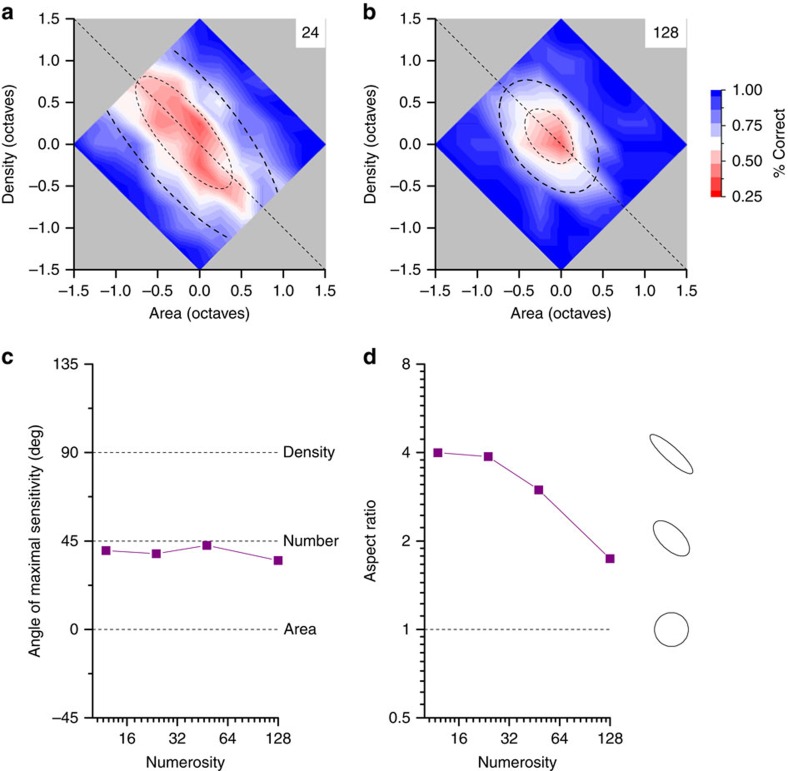
Discrimination boundaries in area–density space. (**a**) 2D psychometric function for measuring thresholds in the area/density space with the ‘odd-one-out' task, for a standard of 24 dots. Per cent correct (pooled across two subjects) is plotted as a function of log area and log density (see heat map at right). The maps show interpolated responses. The raw data were fit with a 2D Gaussian varying between 100 and 33% (chance). The dashed lines show the 50 and 75% performance. (**b**) 2D psychometric function measured with a standard of 128 dots. Conventions as in A. (**c**) The orientation of the short radius (maximal sensitivity) of the best-fitting 2-D Gaussian, as a function of numerosity. The orientation tended to +45° at all numerosities, aligned with the number axis. (**d**) Ratio of s.d. of the long to short radii, as a function of numerosity. For low-to-moderate numerosities the oval was strongly elongated, by a factor of four. Even at the highest numerosity, the oval remained elongated orthogonal to the numerosity axis, with an aspect ratio of 1.7.

The first experiment measured sensitivity in the area–density space with an assumption-free, odd-one-out task. Three stimuli were displayed: two identical standards (40 sq degs, and either 12, 24, 48, 64 or 128 dots), and the oddball which differed from the standard, in a given direction and distance within the area/density space (see ‘Methods' section for details). Subjects indicated which stimulus was different from the other two, without necessarily knowing in which dimension it differed. Figure 2a plots proportion correct responses (pooled across the two subjects) when the sample patches contained 24 dots, and the target patch varied in area and density around that value. The data were well fit by 2D Gaussian functions, whose per cent-correct contours describe ellipses. These are clearly very elongated, and slanted orthogonal to the numerosity axis: this means that under these conditions, the most sensitive dimension is numerosity (the least change was needed in that direction for the standard to be discriminated). Although subjects did not know which dimension had changed, they were far more sensitive to changes in numerosity than to either area or density alone, and spontaneously tended to use that information. Conversely, the poorest performance was when numerosity was kept constant, moving along the main diagonal, although the separate changes to area and density were just as great as when moving along the other diagonal.

To quantify the effect across numerosities we extracted from each map two indexes: the orientation of the short radius of the ellipse (0° is aligned with area, 45° with number, 90° with density) and the ratio of the s.d. of the long- to short-radius. Figure 2b,c plot these for data averaged over two subjects, for numerosities ranging from 12 to 128. Interestingly, for a large range of numerosities (all but 128), the discrimination ellipse is tilted close to 45° and with a high aspect ratio of about four, indicating that numerosity provides the most useful information for the discrimination task. At the highest numerosity used, tilt remains around 45°, but the aspect ratio drops below two, suggesting that numerosity has a lesser advantage for dense stimuli.

### Explicit judgments of density, area and number

The first experiment showed that without instructions, or information about what aspects of the stimuli are changing, subjects spontaneously use numerosity rather than either area or density, questioning the suggestion that area and density are spontaneously used for estimating numerosity. In the second experiment we go further in explicitly asking subjects to make discriminations based on density, area or number (in separate sessions). In this task, we did not score the responses as correct or incorrect, but mapped the proportion ‘more' (number, area or density) on the area–density landscape, and fitted cumulative Gaussian functions. Again, it is the orientation of these functions that indicates the most sensitive dimension.

**Figure 3:**
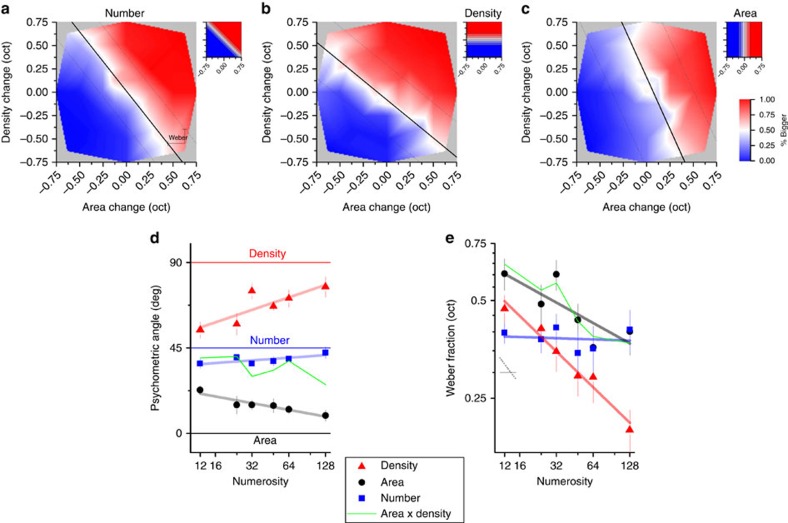
Explicit number area and density judgments in area–density space. (**a**) 2D psychometric functions for explicit number comparisons (which patch appeared more numerous) for a standards 12 dots, plotting per cent ‘more' pooled across six subjects, as a function of log area and log density. The maps are obtained by linear interpolation (see heat map at right for values). The raw data are fitted with a 2D cumulative Gaussian error function, varying between 0 and 100%. The dashed lines show ±1 s.d.; Weber Fraction is the total change needed to attain 84% correct responses. The small insets at right show how an ideal observer would perform, responding correctly to the task. However, the data for all three tasks tended to oriented near the numerosity axis, suggesting that numerosity was used for all tasks. (**b**) Explicit density judgments: conventions as in **a**. (**c**) Explicit area judgments: conventions as in **a**. (**d**) The orientation of the choice axes (deviation from vertical), as a function of numerosity. For the numerosity task (blue symbols), the functions were oriented near +45° at all numerosities (orthogonal to the number axis), suggesting that numerosity provided the primary information for the task. The functions for density (red symbols) were also oriented near +45° at low numerosities, suggesting that density judgments also relied on numerosity. Area judgments (black symbols) were also strongly influenced by numerosity at low numerosities. The green curves show the predictions for numerosity judgments, if they were based on the product of density and area. (**e**) Weber fractions (log (change in area) + log(change in density) at threshold), as a function of numerosity. The lines are best-fitting linear regressions, with slopes of −0.36, −0.21 and −0.01 respectively for density (red triangle), area (black circle) and number. Weber fractions for number remained constant over the range (Weber's law), while density decreased with a slope near −0.5 (square root law). Area also decreased with numerosity. The green curves show the predictions for numerosity judgments, if they were based on the product of density and area. For high numerosities, the predictions are reasonable, but at low numerosities far too high.

Figure 3 shows sample maps for the three tasks for a numerosity of 12 dots, averaged over subjects. If subjects responded as instructed, correctly identifying changes in either number, area or density, the choice planes for the three maps should be aligned orthogonally to those dimensions, as shown in the insets: 45° for number, 90° for density and 0° for area. Surprisingly, however, this did not occur: both density and area judgments showed a strong spontaneous bias towards numerosity. The three maps do not follow their individual ideal-observer predictions, but are all very similar to each other. The numerosity map has a choice plane slanted at 38°, near the predicted 45°. But the slant of density is 54°, nearer 45° than the predicted 90°, and that of area is 23°, about half way between the predictions for number (45°) and area (0°).

Figure 3d,e summarize the data of the discrimination by plotting the two parameters of the decision maps, orientation and width of the 2D psychometric functions, as a function of test numerosity. The orientation of the maps of numerosity judgments is close to veridical across the range of numerosities. However, area and density are both highly biased towards the number axis, indicating that even when asked to make density or area discriminations, judgments are highly biased towards number. Interestingly, at increasing numerosities, the preferred axes become more aligned with the real axes of their dimensions (Fig. 3d). Figure 3e shows how the Weber fraction (essentially the s.d. of the logarithmic cumulative Gaussian function) varies with numerosity. That for number is fairly flat, with a slope of only –0.01, while density decreases with a slope of –0.36, closer to a square root relationship (slope of −0.5). The slope of the regression for area was –0.21, between constant and square root. This result reinforces previous studies showing that numerosity judgments tend to follow Weber's law, while density judgments are better described by a square root relationship^32^,^33^.

The green curve shows the predictions for numerosity (orientation and Weber fraction), if it were based on the product of density and area (see ‘Methods' section for details of the predictions). The simulations predict an orientation near veridical, as observed, but Weber fractions far higher than those obtained, especially at low numerosities. The simulations also predict a decrease in Weber fraction with numerosity, while the data are in fact quite flat. This clearly speaks against the idea that numerosity derives from area and density, at least for reasonably sparse stimuli.

**Figure 4:**
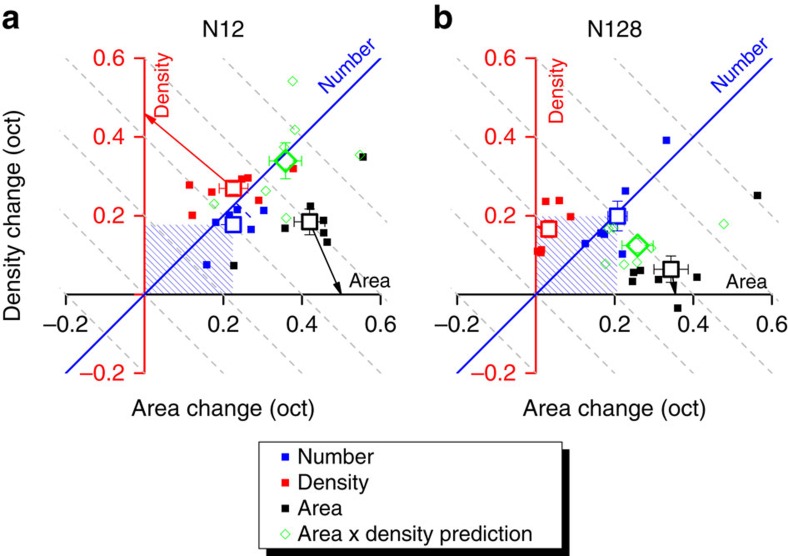
Threshold changes for number density and area discrimination. (**a**) Area and density thresholds (changes required to attain 84% consistent response) for the density, area and number judgments (respectively red, black and blue), for a base numerosity of 12 dots. Dashed lines orthogonal to the numerosity diagonal indicate regions of constant number. Small squares are individual data, large hollow squares (means), error bars are s.e.m. Arrows display projections on the physical axis of area and density thresholds on their respective axis. Green diamonds indicate the predicted thresholds for number if it were calculated from area and density (clearly far higher than actually obtained). (**b**) Like **a**, for base numerosity of 128.

Figure 4 shows individual data for two base numerosities, plotting Weber fractions within the area/density space (large open symbols are group averages). The green symbols show the predictions for numerosity based on the product of density and area. For the higher numerosity (*N*=128), the results are not too far from the predictions, although the predictions tend to be too biased towards the area axis (because area thresholds were worse than density). Nevertheless, the results are not completely incompatible with the notion that number estimates may be derived from the product of area and density, as the mean thresholds lie very close to the predictions. However, the data at more moderate densities (*N*=12) tell a completely different story. Here numerosity judgments clearly do not depend on density and area, as the thresholds are far lower than the prediction from the two components. On the contrary, rather than numerosity being based on density, it would appear to be the other way round, that density judgments are based on number. Even area—a completely different concept—is strongly influenced by number.

In a pilot study we explored the possibility of constructing an arbitrary metric based on the ratio of density and area, which we termed ‘clutter'. This was essentially an ‘anti-numerosity' task, where subjects had to respond more when the stimulus was dense and small, and less when sparse and large. Even experts—including all three authors—failed at this task, even after extensive training. There was no systematic pattern of results, with individual subjects choosing different strategies, and the Weber fractions very large. Even at high numbers the responses did not become close to veridical, suggesting that it is perceptually very difficult to blend area and density, unless the product is numerosity. It appears that the concept of ‘anti-numerosity' does not exist.

## Discussion

In this study we investigated what cues human subjects spontaneously use when judging Numerosity, Density and Area, by varying simultaneously area and density over a wide 2D space. The results show that at low to moderate densities, sensitivity is far higher to numerosity than to density or area. When subjects did not know what aspects of the stimuli were changing (odd-one-out task), they spontaneously based decisions on numerosity. Even when explicitly asked to base their decision of density, observers used numerosity rather than density for discriminations. Area discriminations were also strongly influenced by numerosity. These data speak against the idea that numerosity is extracted from area and density, as sensitivity to numerosity was lower than to either area or density. And when asked to judge density, subjects used numerosity as a primary cue. At high densities the situation changes: density and area seem to be sensed more directly, and numerosity thresholds are consistent with being calculated via density and area. This agrees with previous work showing that texture mechanisms can come into play in numerosity judgments of dense stimuli^32^, and also shows that the technique can, under these circumstances, reveal that numerosity can derive from density and area.

Our data speak clearly to the important point in the dispute as to whether number and density share common resources^16^,^23^,^24^,^25^,^27^,^29^,^31^,^32^. Previous evidence has suggested that number judgments can be influenced by geometrical attributes: for instance, there is a mild positive influence of area^25^ (that is, larger patches appear more numerous). The current data agree with those observations as the plane for numerosity judgments is slightly slanted below 45 degrees (average 38°) indicating that number estimation is mildly influenced by area. It has been argued that this influence parallels that of density, which is also strongly influenced by area^25^. Our data confirm these previous observations but reveal the bigger picture, which is quite different: the influence of area on density in fact results from the density choice plane following the number axis.

Our data also confirm that density judgments are particularly noisy at low numerosities and improve at higher numerosities, tending towards a square root law^22^. Area judgments are also performed rather poorly at low numerosities. As both area and density judgments are performed poorly, they would not make a very useful basis set from which to calculate numerosity, at least at low-mid densities; on the contrary, it seems that information about number is used to aid area and density discriminations.

It may be argued that the low numerosity thresholds do not necessarily imply the existence of direct numerosity mechanisms, but that people are more practiced at making numerosity than density or area judgments, and can therefore do so with more precision. We find this unlikely for several reasons. First, numerosity thresholds at low densities were less than a quarter those of density and area, while learning seldom results in effect-sizes more than a factor of two^35^. Second, as mentioned above, the advantage of numerosity over density is greatly reduced for dense stimuli, where it is almost consistent with the density–area model of numerosity: if it were simply the case that subjects were more accustomed to judging number than density, this should influence the results for dense stimuli as well. However, observers were quite comfortable with the notion of estimating area and density, and did so very well under appropriate conditions. Finally, during the course of this experiment, all observers—particularly the authors—spent many hours training on density and area judgments (far more than in the normal perceptual learning paradigms), and they showed very little improvement. We therefore believe that the low thresholds reflect sensory rather than cognitive mechanisms.

Furthermore, in a series of pilot data we attempted to document what happens if people are asked to mentally combine density and area: we asked them to respond to ‘clutter' an arbitrary, ‘anti-numerosity' dimension. Clutter is not a very intuitive concept, and indeed we found that this type of estimation was almost impossible for observers, even after extensive training. After eight 50-trial sessions of Weber fractions remained very high, and the response criteria variable, with no measureable improvement. This is interesting, as the converse judgment, which can also be considered as a pairing of density and area, is performed veridical, consistently and with low-Weber fractions.

Only at the very high numerosities was the pattern of results consistent with a combination of area and density. In previous papers we have suggested that the cut-off between the two systems occurs where there was a transition from Weber's law to a square root law^22^. With equal area patches, cut-offs were 2 items/degree^2^ in fixation, which decreased to 0.8 and 0.4 items/degree^2^ at 5 and 15 degrees eccentricity^33^. However, that does not preclude the possibility that numerosity mechanisms extend further into the high-density range, with considerable overlap between mechanisms. The current data uses a paradigm in which both area and density change, minimizing possible confounds, and shows that direct number estimation occurs even at 2 items/degree^2^ or more, even in the periphery. The idea that number is derived from density and area may be only to explain those cases at high density.

Our results provide strong evidence that number may be calculated by mechanisms that do not involve density or area. By analogy, we know that velocity can be described as the ratio of space and time, but there is no evidence that the visual system computes each independently and calculates the ratio; on the contrary, there is very good evidence for neural mechanisms specialized for velocity^36^. Indeed, the system seems to be capable of converting time to space via velocity, rather than the other way round^37^,^38^. Similarly, the fact that number can be described as the product of density and area does not mean that the system is obliged to calculate it that way, rather than by more direct means. Interestingly, a recent study by Stoianov and Zorzi^30^ has shown that encoding of number develop naturally during unsupervised learning of a hierarchical generative model of perception. The training concerned only the efficient coding of the sensory data, yet numerosity selectivity emerged as a statistical property of the deepest layer of the model. That coding for number emerges naturally without supervision is consistent with it being a basic property of sensory systems, and that the neural structures subserving this task may be quite simple. For example, Dehaene and Changeaux^21^ have demonstrated that a simple normalization stage with template filters can extract item identity, which can then be fed to a subsequent integrator to estimate numerosity.

One reason why understanding numerosity perception is important is its close relationship with mathematical abilities. Halberda *et al.*^6^ showed that numerosity discrimination predicts math performance in pre-school and school-aged children, confirmed by several other more recent studies (for a recent meta-analysis^39^—but see also^40^,^41^,^42^). Interestingly, however, neither density nor area discriminations predict math performance, either in adults or children^43^,^44^,^45^. This is consistent with the existence of independent mechanisms for estimating number, and potentially important in understanding the links between numerosity and mathematical abilities.

In summary, this study clearly shows that provided items are not packed too densely, estimating numerosity does not require separate estimates of density and area; on the contrary, under many conditions estimating density and area rely on estimates of numerosity. These results point to specialized mechanisms for estimating numerosity, mechanisms completely independent of those involved with density or other seemingly related attributes. These mechanisms probably work at reasonably high levels, after objects have been segregated into ‘countable' quantities. The visual brain has evolved to perceive and interact with a complex visual environment. One strategy for dealing with the complexity is the evolution of brain modules that encode the most salient and invariable attributes of objects and scenes. It is not really surprising that one of these emerging modules should comprise mechanisms that estimate effortless and rapidly the number of items of interest.

## Methods

### Participants

Seven subjects with normal or corrected-to-normal vision participated in this study: 2 of the authors and 5 subjects naive to the goals of the study (3 men, 4 women, 26–38 years old, mean age 28 years). Only two of these participated in the first study, all in the second. Experimental procedures were approved by the regional ethics committee (Comitato Etico Pediatrico Regionale—Azienda Ospedaliero-Universitaria Meyer—Firenze (FI) and are in line with the declaration of Helsinki).

### Stimuli

Stimuli employed throughout all the experiments were clouds of dots with base radius of 3.6 degrees displayed at 12° eccentricity (see Fig. 1). Dots were light- or dark-grey (Weber Contrast 0.4), created by convolving a disc of diameter 20′ with a Gaussian of s.d. 7′. The percentage of light to dark dots was drawn at random for each patch ranging from 20:80 to 80:20 to avoid subjects doing the tasks with either of the subclasses of dots. Patch configurations were calculated offline to meet two conditions: minimum allowed centre-to-centre dot distance was 20′, and aspect ratio of the patches had to be within 0.78:1 (short to longest axis). The standard patch could contain one of six different numerosities (12, 24, 32, 48, 64, 128), but always had a radius of 3.6 degrees (area 40 sq deg.): density therefore ranged from 0.3 to 3.2 dots per deg^2^.

Stimuli were varied using the method of constant stimuli, changing area and density (and consequently number) around the standard for that condition (see Fig. 1). In Experiment 1 (odd-one-out) area and density varied from −1.5 to 1.5 octaves in steps of 0.25 octaves, with the constraint that when both area and density were changing the overall change could not exceed 1.5 octaves. In Experiment 2 (explicit judgment) area and density changed from −0.75 to +0.75 octaves in steps of 0.125 octaves with the caveat that when changing area and density simultaneously the change on each dimension could not exceed at 0.625 octaves.

### Experimental procedures

In the first experiment, designed to measure unbiased thresholds in the area–density space, subjects chose the odd stimulus of three dot clouds, all presented simultaneously at 12° eccentricity at the vertices of a virtual equilateral triangle for 250 ms. Two of the patches (chosen at random) had the same area and density, and comprised the standard. The other (the odd-one-out) differed in either area or density, chosen from the constant stimuli described above. Subjects performed on average 5 sessions of 170 trials each, for a total of about 850 trials per numerosity. In the second experiment, subjects were asked to make an explicit judgment: which of two stimuli had the higher area, density or number (in separate sessions). Stimuli were presented 12° left and right of screen centre simultaneously for 250 ms.

Each subject completed on average 6 sessions of at least 80 trials for a total of 480 trials for each condition yielding a total of approximately 10,000 trials.

### Data analysis

In the odd-one-out experiment, for each numerosity data were analysed by plotting per cent correct responses as a function of the area and density of the odd stimulus. Yield maps like those of Fig. 2, with the abscissa showing change in area and ordinate change in patch density. As the axes are logarithmic, the forward diagonal represents numerosity (area times density), and the other diagonal constant numerosity. Data were fitted with 2D elliptical Gaussian functions:


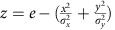
 where *x* and *y* are obtained by clockwise rotation of the area and density space (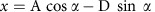 and 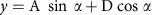). The elliptical Gaussian has five free parameters: orientation of short-radius, widths of the short and long radii and the position of the centre in the area and density space. The orientation of the short radius is the axis of maximal sensitivity as subjects discriminate best when the odd stimulus varied in that direction. The ratio between long and short radii is an index of selectivity of the sensitivity.

In the explicit judgment experiment, for each numerosity and task requirement we plotted the percentage of times the test patch was perceived as ‘more' as a function of area- and density-change. Again, this yields a 2D map of density change plotted against area. Both area and density employed in the analysis were calculated stimulus by stimulus via estimation of the surface covered by the convex hull of the dot cloud.

The subject response maps are then interpolated filling a space spanning from −0.75 to 0.75 octaves and fit with a 2D psychometric cumulative Gaussian function. The fitted function was a one-dimension cumulative Gaussian function operating on an arbitrary axis x obtained via rotation of the area–density space. The function has three parameters: rotation angle, width and offset. The width indicates the amount of change required to go from 50 to 84% ‘more' responses. For choice planes off the area or density axis, the width is expressed as the sum of the changes in area and density required to attain 84% categorization.

### Predicting numerosity from area and density thresholds

To simulate the noise performance of an observer that calculated number from density and area, we assumed that the log of number was obtained as a summation of log area and log density. Performing the simulation in the log of the physical quantities has the advantage that combination of area and density is a simple summation. It can be easily demonstrated that when summing two variables the overall noise is 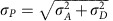 and signal to noise is 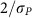. For this reason the threshold lies along the vector composition of the two sources save for an improvement of a factor 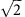. To infer the noise in each judgment we projected the thresholds for area and density task onto their respective physical axis (arrows in Fig. 4).

### Data availability

Data files supporting the figures and the statistical analysis have been made available through the FigShare platform at the following link: https://figshare.com/s/a7773056b2bc7abe5f58

## Additional information

**How to cite this article:** Cicchini, G. M. *et al.* Spontaneous perception of numerosity in humans. *Nat. Commun.* 7:12536 doi: 10.1038/ncomms12536 (2016).
